# Interprofessional collaboration in nursing homes (interprof): development and piloting of measures to improve interprofessional collaboration and communication: a qualitative multicentre study

**DOI:** 10.1186/s12875-017-0678-1

**Published:** 2018-01-11

**Authors:** Christiane A. Müller, Nina Fleischmann, Christoph Cavazzini, Susanne Heim, Svenja Seide, Christina Geister, Britta Tetzlaff, Andreas Hoell, Jochen Werle, Siegfried Weyerer, Martin Scherer, Eva Hummers

**Affiliations:** 1 Department of General Practice, University Medical Center Göttingen, Humboldtallee 38, 37073 Göttingen, Germany; 2 Faculty V – Health, Religious Education and Social Affairs, University of Applied Sciences and Arts, Blumhardtstraße 2, 30625 Hannover, Germany; 3 Department of Primary Medical Care, University Medical Center Hamburg-Eppendorf, Martinistraße 52, 20246 Hamburg, Germany; 4 Psychiatric Epidemiology and Demographic Change, Central Institute of Mental Health, Medical Faculty Mannheim/Heidelberg University, J5, 68159 Mannheim, Germany

**Keywords:** General practitioners, Interdisciplinary communication, Nursing homes, Primary health care, Physician-nurse relations, Qualitative research, Residential facilities

## Abstract

**Background:**

Given both the increase of nursing home residents forecast and challenges of current interprofessional interactions, we developed and tested measures to improve collaboration and communication between nurses and general practitioners (GPs) in this setting. Our multicentre study has been funded by the German Federal Ministry of Education and Research (FK 01GY1124).

**Methods:**

The measures were developed iteratively in a continuous process, which is the focus of this article. In part 1 “exploration of the situation”, interviews were conducted with GPs, nurses, nursing home residents and their relatives focusing on interprofessional interactions and medical care. They were analysed qualitatively. Based on these results, in part 2 “development of measures to improve collaboration”, ideas for improvement were developed in nine focus groups with GPs and nurses. These ideas were revisited in a final expert workshop. We analysed the focus groups and expert workshop using mind mapping methods, and finally drew up the compilation of measures. In an exploratory pilot study "study part 3" four nursing homes chose the measures they wanted to adopt. These were tested for three months. Feasibility and acceptance of the measures were evaluated via guideline interviews with the stakeholders which were analysed by content analyses.

**Results:**

Six measures were generated: meetings to establish common goals, main contact person, standardised pro re nata medication, introduction of name badges, improved availability of nurse/GP and standardised scheduling/ procedure for nursing home visits. In the pilot study, the measures were implemented in four nursing homes. GPs and nurses reviewed five measures as feasible and acceptable, only the designation of a “main contact person” was not considered as an improvement.

**Conclusions:**

Six measures to improve collaboration and communication could be compiled in a multistep qualitative process respecting the perspectives of involved stakeholders. Five of the six measures were positively assessed in an exploratory pilot study. They could easily be transferred into the daily routine of other nursing homes, as no special models have to exist in advance. Impact of the measures on patient oriented outcomes should be examined in further research.

**Trial registration:**

Not applicable.

**Electronic supplementary material:**

The online version of this article (10.1186/s12875-017-0678-1) contains supplementary material, which is available to authorized users.

## Background

Older people will increasingly live in nursing homes over the coming decades in Germany [1] and most Western European Countries. Nursing home residents represent a very dependent, vulnerable and frail subgroup of the elderly should receive the best medical care. It is known that improvement of interprofessional collaboration and communication could contribute to better patient oriented outcomes generally [2] as well as in the nursing home setting [3]. On these grounds we explored the situation of medical care in the nursing home setting in Germany with the aim to compile measures for better collaboration and communication between GPs and nurses. These measures were then tested for acceptance and feasibility in an exploratory pilot.

Organisation of medical care in German nursing homes differs from many other countries. Here, mainly self-employed community based GPs provide nursing home visits to the residents who are part of their practice clientele [4]. On average, 23 physicians (most of them GPs) provided care to a single nursing home in a German study [4], resulting in a multitude of constellations of GPs and nurses in collaboration. Nurses and nurse aids work in the nursing home mostly in part time, nurse practitioners do not exist in the German Health System [5]. Not all GPs perform home visits to nursing home residents. Provision of care to nursing home residents is generally seen as a professional obligation which implies high emotional demands and is often considered burdensome [6]. Nurses in German nursing homes are solely responsible for the basic nursing care of the residents; a physician must explicitly delegate all procedures concerning medical care [7].

Information on quality of care in nursing homes is rare. Provision of medical care by general practitioners was considered as “sufficient” in a report of the statutory health insurances in 2009. Here on average one visit per quarter was provided by GPs. However an oversupply of psychotropic drugs and antidepressants as well as an undersupply of antidementives was assumed [8]. The quality of nursing care in nursing homes can only partly be assessed on basis of the three-year reports of the Medical Service of the Health insurers; data is mainly collected from inspection of resident files. Quality of care has been found improved in many fields as compared to the last report three years earlier, whereas management of pain and medication were aspects with room for improvement [9]. Additionally a recent Health Technology Assessment report indicated that German nursing home residents suffering from dementia or diabetes are over- or under-supplied in some aspects of their medical care because of inadequate documentation, prescribing, and insufficient intra- and interprofessional communication [10].

In general, issues with communication are thought to be responsible for most mistakes in medical care [11, 12]. The Advisory Council on the Assessment of Developments in the Health Care System recommends to find new forms of cooperation of health professionals to provide health care more efficiently and effectively [13]. Recommendations for better interprofessional collaboration in the nursing home setting were published by various German organisations and stakeholders [14]; and cooperation agreements are now required by law [15].

In Germany there are a few model or concept projects exploring alternative organisational structures and new forms of collaboration to improve interprofessional collaboration in nursing homes [16–18]. Effects seem to be positive, although scientific evaluation is rare [10, 19]. To date, only a few German studies have qualitatively explored the perspectives of partners involved in nursing home care, and none have done so to develop specific measures for better cooperation [20–22]. We developed measures to improve interprofessional collaboration and communication between GPs and nurses in nursing homes in a qualitative bottom-up action research process. Finally the measures were implemented in an exploratory pilot study in four nursing homes for a three months period and evaluated qualitatively with regard to acceptance and feasibility.

## Methods

### Design of the study

**Fig. 1:**
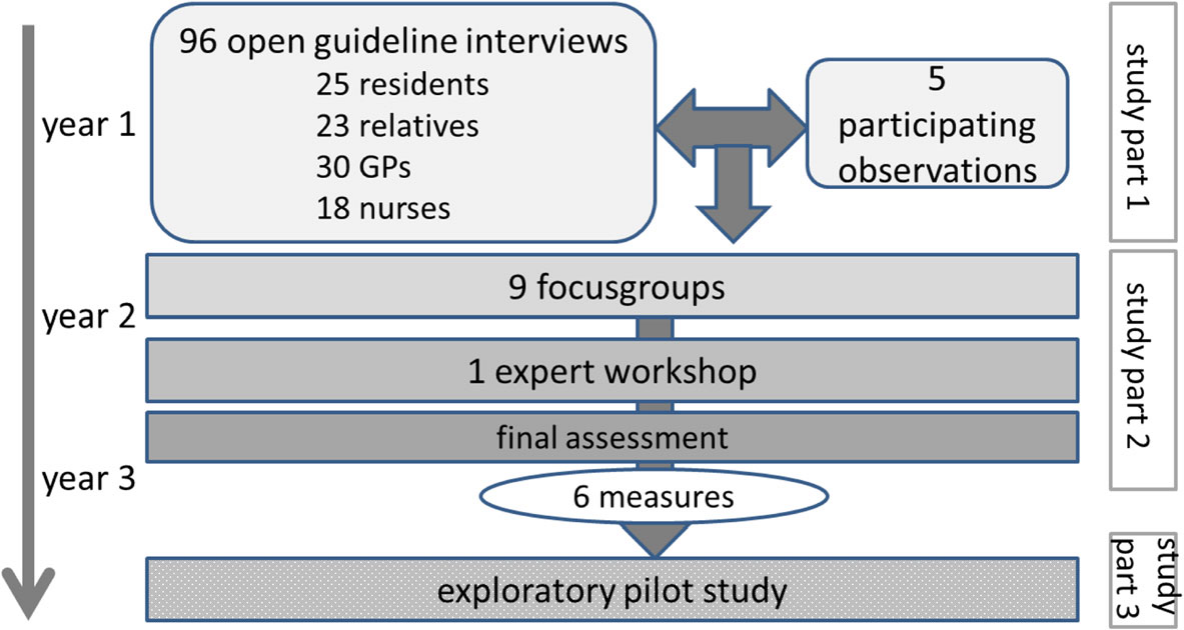
Study design

The interprofessional research team developed the measures in a multistep process (fig. 1) which is the main focus of this article. Additionally the measures were tested in a pilot study for a three months period. The researchers were located in the three study centres in Göttingen, Hamburg and Mannheim.

#### Study part 1 “exploration of the situation”

A: Open guideline based interviews, participating observations of GPs’ nursing home visits.

#### Study part 2 “development of measures to improve collaboration”

B: Single- and interprofessional focus groups, guidelines based on results of A.

C: Expert workshop (various stakeholders and other participants) guideline based on results of B.

D: Final assessment: Selection of measures on the basis of steps A to C.

#### Study part 3 “exploratory pilot study”

Implementation and evaluation of the developed measures in four nursing homes for a three months period.

### Research team, reflexivity, framework

The research team consisted of a gerontologist (AH, male), an occupational therapist with master of science (BT, female), a sociologist (CC, male), a medical doctor, qualified radiologist and master of public health (CM, female), two professors of general practice (EHP, female; MS, male), a sports scientist (JW, male), a professor of epidemiology (SW), a qualified nurse, nursing scientist and master of public health (NF, female), a literary scientist (SH, female), a medical statistics student (SS, female) and two medical students (female, no authors). CG, female, a qualified nurse and professor of nursing science trained the research team in methodological aspects and supervised the study. CM, EHP, SH and CG, as well as AH and JW, knew each other before the study start. The other researchers also got to know each other following the initiation of the study. CG, EHP, CM, CC, BT and SH were already experienced in qualitative methods. The theoretical framework used is Campbell’s “Framework for design of complex interventions to improve health” [23], consisting of one preclinical and four clinical phases: 1) modelling, 2) exploratory trial, 3) definitive randomized controlled trial and 4) long term interpretation. Our study comprises an exploration of the situation (part 1) and the development of measures (part 2) in a multistep qualitative process which could be collated to phase 1 (modelling). In part 3 we piloted the developed measures, which could be compared to phase 2 (exploratory trial) of this framework. The study was conducted between March 2012 and July 2015.

### Data collection and analyses

#### Study part 1 “exploration of the situation”

##### A: Open guideline interviews and participating observations

The research question and proposal for this study had been developed based on the national and international literature about the topic. Interview guidelines according to the research questions were developed by the research group (interview guidelines in Additional file 1). We intended to capture hidden, implicit assumptions of the participants. In addition to the exploration of the respective perspectives on medical care in the nursing home setting, we were interested in the interprofessional interactions. Interviews should be conducted in an open manner without any presuppositions from side of the interviewer. We tested the interview guidelines in interviews with relatives and medical and nursing colleagues [7]. We recruited nursing homes via postcodes and existing relationships to the three study centre institutes. GPs were contacted via the research networks of the participating institutes, additionally via postal codes or personal relationships. Purposive sampling was mainly used to recruit participants, when we realized that the initially planned theoretical sampling was not feasible. Purposive sampling was used to easily recruit GPs and nurses. However, for relatives and residents it proved to be more difficult and after an initial period of purposive sampling, we had to include all persons willing to participate. Specifics of purposive sampling were for nursing homes: size, location, funding organization; for nurses and GPs: age, sex, years of working experience; for residents and relatives: age, sex, length of stay of nursing home resident in nursing home. In study part 1 we recruited 18 nursing homes in the study centres and surroundings: 6 in Göttingen, 4 in Hamburg and 8 in Mannheim. The size of the nursing homes differed from less than 40 to more than 200 residents. We only included residents older than 65 years who had lived in the nursing home for at least 3 months. Persons under supervision as well as those with high-degree cognitive impairments, severe physical infirmity and insufficient command of German were excluded.

The management of the participating 18 nursing homes invited nurses, residents and relatives as interview partners. A few residents were additionally invited by their GP.

After obtaining informed consent, trained members of the research team conducted face-to-face open guideline interviews [24] with 30 GPs, 18 nurses, 25 nursing home residents and 27 residents´ relatives. The interviews took place in nursing homes, GP practices, in the interviewee’s home or in rooms of the involved research institutes. In one study centre, the researcher interviewed a convenience sample of GPs known from previous interactions, in the other study centres researchers recruited most interviewees de novo. The researchers’ assumptions were not shared with interviewees. Memos were taken after the interviews. Interviews lasted 6–68 min (residents), 26–77 min (nurses), 20–58 min (relatives) and 19–46 min (GPs). Privacy was provided during the interviews, as they were conducted under four eyes. Exceptions were 4 double interviews, where two relatives of one resident were present at the same time (thus, 23 interviews with a total of 27 relatives).

Interviews were audio recorded, transcribed and re-checked against the audio record; the interviewee names and other identifiers of persons and locations were replaced with pseudonyms. Interviews were analysed according to the first steps of grounded theory methodology by initially creating open codings by constant pairs of two researchers with different professional backgrounds (residents: CM and CC, nurses: NF and AH, GPs: BT, JW, NF, relatives: CC and medical doctoral student) [25]. MAXQDA 10 (Qualitative Data Analysis Software) was used to manage the codings. Superordinated memos for each stakeholder were devised afterwards focusing on obstacles and opportunities of interprofessional collaboration as well as interviewees´ perspective. Findings were integrated into the focus group guidelines in study part 2. Parallel to the study process presented in this paper the researchers also continued analysing the material in depth generating axial codings (using the paradigm model) and selective coding to compile models for the different stakeholder. The in depth exploration of the material took more time than the development of the measures. First publications on GPs´ and nurses´ perspectives on nursing home visits are already available [26, 27].

Five GP nursing home visits were observed passively by trained researchers (CC and NF), focusing on the interaction of all participants [28]. During the visits short memos were taken. Immediately afterwards an extensive observations protocol was completed. Insights from the participatory observations were integrated into the analyses of the interviews.

#### Study part 2 “development of measures to improve collaboration”

##### B: Focus groups

Three focus groups were conducted in each of the three study centres (facilitators: AH, BT, CC, CM, JW, NF): monoprofessional focus groups with either nurses or GPs and a third GP and nurse interprofessional group. The group size varied between four to eight participants attending the single professional groups; the interprofessional focus groups contained eleven participants. Altogether 34 nurses and 34 GPs participated. Participants were recruited by a procedure similar to the guideline based interviews; a few of them had been interviewed previously. Strengths and weaknesses of the current collaboration were discussed in the monoprofessional focus groups; initial ideas for measures were drafted. Based on the findings the guideline for the interprofessional focus groups was developed. In the interprofessional focus groups, the suggestions for better collaboration and communication were again discussed and then prioritised. The focus groups were video recorded in two study centres, in the third they were audio-recorded. All focus group discussions were transcribed. Analyses were performed using mind mapping methods by all facilitating researchers in teams of at least two persons [29]. We used the mind mapping approach as it is a pragmatic approach based on a summarising, structured analysis of the data. Findings were condensed by the research team in Göttingen and again discussed in telephone conferences with all involved researchers.

##### C: Expert workshop

In the one day expert workshop in Göttingen (facilitators CM and SH) participants with different professions took part: a nursing home director, a nursing home manager, a quality manager, head nurses, GPs partly with a geriatric focus who frequently visited nursing homes, a nursing home doctor from a German concept project, a communication expert, a professor of nursing science, a public health specialists, a sociologist and a relative representative. Based on the findings from the focus groups, measures for better collaboration/communication were discussed, and strategies for recruitment and implementation were developed. The expert workshop was video-recorded. Again mind mapping methods were used [29], analyses were performed by CC, CM, NF under the supervision of CG and EHP. Findings were sent back to the participants for comments and corrections.

##### D: Final assessment

The core research team (CC, CG, CM, EHP, NF) finally selected the measures through several meetings, taking into account the findings from the various steps (A-C) with the main weighting on the expert workshop.

#### Study part 3 “exploratory pilot study: Implementation and evaluation of the developed measures”

After the development of the measures we tested them for feasibility and acceptance in a pilot study. Findings should indicate how GPs and nurses judge the usefulness of the measures in practice.

We invited 20 nursing homes in Göttingen and the surrounding areas; four agreed to participate. They were run either as non-profit organisations or private companies, their size varied from small to large (exact numbers cannot be provided to preserve anonymity). Two of the homes have already been involved in the development of the measures. The measures to be adopted, their implementation and the recruitment strategy for GPs were decided in preliminary meetings with researchers and nursing home management. During a launch meeting in every nursing home the chosen measures were discussed by the nursing home management, head nurses and nurses, GPs and two researchers, and were adapted to individual nursing home’s needs until everyone gave consent. The finalised measures were introduced into the nursing homes for a three months period (Table 1). In the largest nursing home, most GPs (*n* = 7) and nurses (*n* = 6) were involved, in the other three nursing homes, one GP and, one, two, or three nurses took part, respectively. This constellation of involved GPs and nurses concerned the care of 20 residents. After three months, eleven GPs and twelve nurses were interviewed in brief face-to-face guideline [24] based interviews with regard to feasibility and acceptance of the implementation of the measures (interview guideline in Additional file 2). In addition we recorded some demographic data as well as a few standardised questions with regard to perceived reduction of nursing home visits and estimated benefit for the residents. Only one resident and one relative were willing to be interviewed. Content analysis of the interviews were performed using MAXQDA 10 in a step wise process consisting of i. a. case related summaries, coding according to the category system on basis of the interview guideline, comparing of the codes of the two analyzing researchers, adjustment and later merging of the codes into one dataset, paraphrasing of coded text passages [30].

**Table 1:** Selection of measures to improve interprofessional collaboration/communication in nursing homes (pilot study)

Measure defined by research team	Measures selected by nursing home management			
	*Nursing home A*	*Nursing home B*	*Nursing home C*	*Nursing home D*
Meetings to agree on objectives	–	X	X	–
Main contact person nursing home/GP’s practice	–	–	X	–
Standard pro re nata medication	X	–	–	–
Introduction of badges	X	X	X	X
Accessibility of GPs and nurses	X	X	X	X
Date arrangement and procedure for home visits of residents	–	X	–	X

## Results

### Study part 1 “exploration of the situation”

#### A: guideline interviews, content of superordinated memos after open coding

**Residents** themselves made little experiences with interprofessional communication and cooperation beside the home visits and showed low interest into interprofessional interactions between GPs and nurses. Residents had little opportunity to observe interactions between GPs and nurses due to spatial conditions.

**Relatives** were also rarely present when interprofessional cooperation took place; therefore this topic was of little relevance to them. They receive information on medical care from nurses or GPs separately. They described interprofessional cooperation and communication mainly based on assumptions.

**Nurses** considered interprofessional cooperation as useful when they perceived their interactions with the GP as beneficial for the residents. In contrast, a lack of respect, divergent assessment of symptoms, unclear instructions for therapy and stressed GP behaviour were perceived as failed cooperation. Suggestions to improve cooperation were based on better interactions: Nurses themselves suggested more responsibility in the context of medication, better linkage of the documentation systems between nursing homes and GP practices and faster responsiveness of GPs in acute situations. To improve the relationship with GPs, nurses requested more mutual respect and knowledge/ appreciation of their profession by the GP. Finally, the nurses suggested there be a nursing home physician and a pharmacy co-located in the nursing home.

From the **GPs’ perspective**, good cooperation implied agreements regarding timing, meetings and shared responsibility. They criticised inadequate implementation of instructions, lack of information flow and a high turnover of staff. For a better interprofessional cooperation, the interviewees also suggested organisational improvements including better coordination before contacting the GP, a cross-shift contact person and again a linkage of the documentation systems. GPs had similar suggestions for better communication: mutual respect, the establishment of a culture of openness to raise issues, the delegation of responsibility, key service training, therapeutic team meetings/ case conferences and informal meetings. Additionally, a rationalisation of processes, also the introduction of nursing home physicians and better qualified nurses were mentioned.

### Study part 2 “development of measures to improve collaboration”

#### B: Focus groups

Eleven aspects for better cooperation could be extracted in the single profession focus groups: **accessibility**: 1) reliable accessibility of the GP via telephone, 2) cross shift contact person in the nursing home; **allocation of tasks**: 3) resident oriented team meetings 4) definition and differentiation of tasks and responsibilities; **GP nursing home visits**: 5) announcement of visits or fixed times for visiting 6) nurse company during GP visits including pre-preparation and post-processing; **relationship level**: 7) more appreciation and equality 8) establishment of a communicative culture; **transparency**: 9) “round table” 10) more transparency of processes 11) offer of continuing education.

In the interprofessional focus groups, these suggestions were ranked and discussed. Finally we could identify the following ideas for further discussion in the expert workshop (Table 2):

**Table 2:** Ideas for better cooperation (findings from interprofessional focus groups)

Ideas for better cooperation	examples
Mutual accessibility	• use of faxes: obligatory processes, standardised forms, notation on urgency • establish telephone consulting hours for the GP • acute telephone calls: availability of mobile number of GP • contact nurse in the nursing home: competent, informed, skilled, cross-shift, one mobile phone per living area (residential unit)
Fix date for GP’s visit	• fixed date: nursing home sets date, fixed weekday, timing of a 30 min period of time • announcements and arrangements: via fax, timely cancellation of date by GP • central telephone number in nursing home for information transfer
Preparation Company during GPs’visits Postprocessing	• prior to visit: communication via fax, nurse compiles and prepares records, (standardised) prioritisation of inquiries • preparation: chart round by GP and nurse • resident visit: preferably with nurse, standardised documentation • postprocessing: realisation in the responsibility of the nurse • soft skills: reliability, trust, openness, agreements, sufficient time
Transparency Definition of tasks	• regular exchange of medical information • periodical assessment of diagnosis and therapy (GP) • reliable/competent reporting (nurse) • name badges • clarification of responsibilities and expectations • information about processes in the nursing home and GP practice
Appreciation and respect	• mutual respect, trust, tolerance of mistakes • establishment of a communication culture • acknowledgement of nurse /GP competencies (feedback, enough time, listening, GP asks for suggestions from nurses) • to be on equal level, provision of patient care in partnership

#### C: Expert workshop

The experts revisited and rigorously discussed the measures extracted from the focus groups. Although they were instructed to prioritise the measures, to discard those of less importance and to add new themes all measures preset in the focus groups were considered as equally relevant and none were overruled. Additionally, the experts gave detailed advice for the recruitment of participants and developed a sequence of steps for the implementation of measures in nursing homes.

#### D: Final assessment by the research team

Based on the findings from the expert workshop, study part 1 and 2 and national and international literature we compiled six measures likely to have a positive impact on the interprofessional collaboration/communication between GPs and nurses within three months.

### Measures to improve interprofessional collaboration and communication

1. Meetings to establish common goals

The GP, a qualified nurse and a resident with a relative (optional) discuss long term goals: “What goals do we have? How can we proceed together? Who is responsible? When will we reassess the goals?” The main fields considered include: mobility, nutrition, social integration, individual medical features. A small laminated flow chart supports the interprofessional communication. The goals are recorded in the nursing documentation; a copy is provided to the GP.

2. Main contact person in the nursing home and GP practice

A qualified nurse ensures the communication with the internal team and with the GP face to face and / or via telephone. The nurse should be experienced in nursing home care, informed about the residents and the current care standards, competent with regard to care skills and communication. Finally she fulfills tasks responsibly. A deputy will be nominated in the case of absence. In the GP practice a practice assistant acts as the main contact person for the nursing home(s). The practice assistant prioritises the requests of the nursing homes with regard to their importance and urgency. The practice assistant and the main contact nurse share same competencies.

3. Standard pro re nata medication

A specific and appropriate pro re nata medication reflecting the resident’s health situation is established in meetings to agree mechanisms that prevent unnecessary telephone calls and faxes. If particular symptoms occur a form helps to decide about the medication, the dose and the dosing schedule. Moreover the form states what other factors have to be considered and when the doctor needs to be informed. The pro re nata medication form is added to the nursing documentation, a copy is provided to the GP.

4. Introduction of name badges

The main contact nurse and GP wear name badges when working in the nursing home. They are responsible for name badge storage and carriage.

5. Improved availability of the nurse/GP

Availability corresponds to accessibility via telephone and via fax. The main contact nurse is reachable via telephone or an answering machine takes the call, but responses are prompt. GPs may provide their private mobile number to the main contact nurse. During phone conversations, both parties are to behave in a respectful and constructive manner. An accessible and functioning fax machine has to be provided in the nursing home. The sender has responsibility to ensure that an answer is received. A fax form with different sections can be used: a) resident’s personal data, name of the contact nurse and nursing home, b) the request itself, information on urgency and an optional read receipt acknowledgement, c) GP response and optional read receipt acknowledgement, d) comment from the nurse regarding implementation of advice. The form is stored within the nursing documentation/GP patient records.

6. Standardised scheduling and procedure for nursing home visits to residents

Dates for home visits are announced by the GP at least two days in advance specifying a two hour time slot. Ahead of the GP visit, the main contact nurse prioritises the demands of residents/ nurses, the GP contacts the main contact nurse, both review the requests and the main contact nurse offers her company during the resident round. During the visit (with or without the nurse) the GP makes notes and files them with the nursing documentation. Afterwards the GP informs the main contact nurse directly or makes clear that notes are filed with the nursing documentation.

The overall development of the aspect “fax form” of measure 5 (improved availability of nurse/GP) has been described in detail in the Additional file 3, Additional file 4, Additional file 5, Additional file 6, Additional file 7.

### Study part 3

#### Exploratory pilot study

The findings of the content analyses of the interviews indicated that the measures were overall evaluated positively; only in one nursing home changes were reported solely by the nurses, but not by the GP involved. Most of the interviewees (GPs and nurses) reported positive experiences with the implemented measures except for the “main contact partner” measure. This was implemented in only one nursing home by one GP and two nurses and was not considered useful, as cooperation had already been good. The other five measures were generally easy to implement and well accepted. Even small changes such as the wearing of name badges (in all four nursing homes) was judged to have a good impact and resulted in a perceived improvement in communication and collaboration. Date arrangements for home visits were perceived differently: nurses preferred fixed advanced GP visiting times in order to prepare for the visit adequately; GPs complained about the reduced flexibility, but also saw advantages for their workflow. Both parties thought that residents benefited from the “standardised scheduling and procedures for nursing home visits of residents”. The cooperation between GPs and nurses improved. Nurses emphasised feeling more valued with regard to their competencies. More time was needed for the more intensive collaboration (mainly meetings to establish common goals), particularly in the beginning of the implementation. However the staff learned to structure the collaboration more effectively and found that reflecting on their interactions was also a positive effect.

Asked if a change in frequency of nursing home visits had occurred, all but one GPs reported no difference whilst five of eleven nurses claimed that visits took place more often. Nurses estimated that measures were of moderate to big influence on residents´ medical care, GPs thought effects to be small to moderate. Asked for the relation of effort and benefit of the measures (benefit =0, effort =1) both groups decided on more benefit (mean: nurses 0,35, GPs 0,31).

The one resident and relative however did not perceive a change in medical care or interprofessional collaboration/communication.

## Discussion

In a thorough bottom-up action research process we developed six measures to improve collaboration and communication between GPs and nurses in nursing homes. Measures were implemented in four nursing homes over a three months period. An exploratory qualitative evaluation showed mainly positive results with regard to feasibility and acceptance.

The project interprof and its results are unique in Germany. Other projects only described the current situation using content analyses [20] or grounded theory [22], without developing measures or attempting to act on their findings. Another recently published large German study [21] provides suggestions for better cooperation in nursing homes through a mixed-methods approach; they used semi-quantitative questionnaires and focus groups as sources; data was analysed using pragmatic techniques focusing on “direct comments”. From our methodological process, especially the interview analyses, we could also capture hidden implicit assumptions in the narratives of the interviewees. In the study by Karsch-Völk, the most frequently stated suggestions for better cooperation were improved communication (9%), higher remuneration for home visits to nursing homes (7%), regular visits (5%) and less bureaucracy (5%) [21]. These results agree partially with much older data from a postal survey of physicians in Berlin, where 48.7% of respondents suggested a better remuneration for their visits, 23.8% argued in favour of reducing administrative processes, 23.5% supported an increase in nursing home staff numbers and 20.9% recommended improving communication [31]. None of these projects were designed to be representative. Nevertheless, our six measures encompass some of the aspects mentioned in the above publications such as better communication (established contact partner, recognised processes before and during nursing home visits, meetings to establish common goals) and regular visits (timely notice). Some of the other suggestions were discussed intensively in the focus groups or were recorded from the interviews (remuneration, more personal in nursing homes), but could not be incorporated into the measures, as they require profound changes of the German healthcare and nursing care system. Changing these higher-level organisational and political issues is clearly beyond the scope of our study, though our measures contained some suggestions regarding local bureaucracy (fax form, standardised pro re nata medication, processes before and during nursing home visits).

Acceptance and feasibility of our measures were preliminarily confirmed in our explorative pilot study. In another German project, nursing home nurses received an educational intervention focusing on nurse-physician communication; multipliers were educated to pass on their knowledge and skills to their colleagues. Following this, nurses experienced a more structured communication with GPs including the definition of goals and nursing assistants also felt more self-confident when communicating with physicians [32]. In a recent US study trainees of several healthcare professions and medical specialties in their first year, conducted individual interviews with nursing home residents prior to a weekly interprofessional meeting, in which they discussed individual residents´ health status and developed interprofessional care plans [33]. Similar to the findings of our pilot study, these students considered team meetings (pilot study: meetings to fix common goals) to improve the quality of care, though direct effects on resident outcomes were not evaluated. In the ELDER project, health care workers but not GPs took part in a three year curriculum to improve interprofessional communication and collaboration in the care of older adults [34]. Teamwork and communication knowledge did not significantly rise between pre- and post-testing, and participants working in long-term care felt time constraints prevented them from collaborating with other professions, although they wished to do so [34]. Time constraints were also mentioned in the interviews of our pilot study. The GPs generally considered interventions as time consuming, although they realised the advantages and benefits of the implementation. A recent review found that interventions in nursing homes are more effective for resident health if resident’s GP and/or a pharmacist are involved. Moreover, improved team communication and coordination had a positive impact [3]. Following this work, we will now (April 2017) start to implement the measures in a randomised controlled trial to assess effects on residents´ health.

The strength of this study is the stepwise bottom –up approach to develop measures to improve collaboration. Measures were shaped by the real experience, needs and wishes of all stakeholders. Moreover, the composition of the interprofessional team which conducted the interviews and focus groups in the three centres and the analyses and discussion of the data in mixed professional teams contributed to the diversity of findings. Our approach, as described in our study protocol [7], is of high rigour according to Guba and Lincoln [35].

### Limitations

In this study interprofessional collaboration between two professional groups was evaluated: GPs and nurses. We did not involve other allied health professions, e.g. physiotherapists, behavioural therapists, psychologists or social workers. While being aware of the important role of these medical professionals in residential care, we felt it important to focus on the GPs and nurse interaction in this environment. Interprofessional collaboration between all medical staff is important and therefore should also be explored with further research and interventions.

We moreover should disclose some methodological weaknesses: it was not possible to perform theoretical sampling due to organisational reasons (transcription time needs, teamwork of different study centres, difficulties of recruitment in nearly all groups). Given this, we mainly performed purposive sampling for the recruitment of interview partners. However, we reached saturation in the data of all interviewed stakeholder groups later on.

Similarly, participatory observations of nursing home visits were also challenging to organise. Several attempts failed due to the limited and often spontaneous visits by GPs. In the end, only five observations were conducted over a much longer period than initially planned. However, aspects of the observed interactions influenced the analyses of the interviews.

Residents or relatives were not integrated into the focus groups for the development of the measures; this was not part of the proposal. With hindsight we should have endeavored at least to include relatives and invite them to additional focus groups. We did though manage to invite a relative representative to the expert workshop, who gave valuable input.

It can be also criticised that the measures are neither really new nor extensive and could have been compiled using common sense. We agree that the measures appear quite straightforward. However, they have been identified and prioritised by key professional staff groups and experts. In addition, nursing homes chose varying combinations of measures, as some had not been implemented before.

Moreover, the pilot study only provides an impression of the acceptance and feasibility of the implementation of the measures in the nursing homes and cannot provide robust data. The findings give an orientation of the usefulness of the measures and possible barriers during the implementation process. Moreover, the numbers (implemented measures, nursing homes, participating nurses and GPs and residents/relatives) were small. Another weakness is the low number of residents and relatives that participated in the evaluation of the pilot study. Unfortunately, most residents were not able or willing to be interviewed and despite significant effort on our part we could find only one relative of the 20 residents who was willing to participate. The resulting information from the resident and relative interview was therefore insufficient.

## Conclusions

Based on our qualitative exploration and bottom-up stepwise action research approach we were able to take into account contributions of all those involved and include additional expert knowledge to develop measures to improve interprofessional collaboration. The developed measures were simple to implement and generally received positively in the exploratory pilot study. The measures can easily be transferred into the daily routine of other nursing homes, as no special protocols or projects are required. The professional staff involved was confident that residents would benefit. Although the impact of the measures on nursing home residents and their medical care was not part of this study, it will be the next step to evaluate a broader implementation concerning resident related outcomes.

## Additional files

Additional file 1: Interview guidelines study part 1. (PDF 192 kb)

Additional file 2: Interview guideline study part 3. (PDF 175 kb)

Additional file 3: Interview quotations availability fax. (PDF 147 kb)

Additional file 4 Monoprofessional focus groups summary accessibility. (PDF 176 kb)

Additional file 5: Interprofessional focus groups summary results fax. (PDF 189 kb)

Additional file 6: Expert workshop results fax. (PDF 11 kb)

Additional file 7: Fax form. (PDF 197 kb)

## Abbreviations

GP: General practitioner
