# Supplementary material for: Interprofessional collaboration in nursing homes (interprof): development and piloting of measures to improve interprofessional collaboration and communication: a qualitative multicentre study
Source: BMC Fam Pract. 2018 Jan 11;19:14. doi: 10.1186/s12875-017-0678-1 (PMC5765653; doi:10.1186/s12875-017-0678-1)
Supplement: Supplementary file 1 — Interview guidelines study part 1. (PDF 192 kb) [file 12875_2017_678_MOESM1_ESM.pdf]

Additional file 1

Interview guidelines study part 1

Interview guideline GPs (first published in: Fleischmann N, Tetzlaff B, Werle J, Geister C, Scherer M, Weyerer S, Hummers-Pradier E, Mueller CA: Interprofessional collaboration in nursing homes (interprof): a grounded theory study of general practitioner experiences and strategies to perform nursing home visits. BMC family practice 2016, 17:123.)

Narrative of a typical home visit in nursing homes	You have been working here in a nursing home for some time. Today we are interested in your experience of how a nursing home visit is usually carried out. Tell us about typical situations as well as positive and negative experiences during the visits. Can you describe exemplary situations?
Description of the last GP visit	Could you please recall your last home visit to a nursing home resident? How did this particular visit go? Please describe the visit in detail.
Experience with areas of responsibility and distribution of tasks	We are interested in the distribution of tasks in a nursing home. What are your tasks and responsibilities, and what are the tasks and responsibilities of the nursing home staff?
Ideas and vision about the ideal care in a nursing home	Give your imagination free reign. How do you imagine ideal medical care in a nursing home would be provided? What would you like to see; also in the case if your parents were residents there? What do you think the nurses would expect? What process might be optimal for the nurses? And what processes would be best for the residents and what would they likely prefer?

Interview guideline nurses (first published in: Fleischmann N, Geister C, Hoell A, Hummers-Pradier E, Mueller CA: Interprofessional collaboration in nursing homes (interprof): A grounded theory study of nurse experiences of general practitioner visits. Appl Nurs Res, in press)

**Narrative of a typical work day/ home visit in nursing homes**	You have been working in a nursing home for quite a while. We are interested in your everyday work experience. How does a regular day look for you? What are typical situations? What are the positive and negative experiences during your daily work? Can you describe exemplary situations? How does the medical care proceed usually?
**Description of the last GP visit**	Please remember the last home visit of a general practitioner in the nursing home. How was it in this particular case? Please describe the exact process.
**Experience with areas of responsibility and distribution of task**	We are interested in the distribution of tasks in a nursing home. What are your tasks and responsibilities and what are the tasks and responsibilities of the general practitioner?
**Ideas and phantasies about the ideal care in a nursing home**	Let your imagination run wild. What would be the ideal medical care in a nursing home for you? What would you like to see there? What do you think would the general practitioner expect? And what processes would be optimal for the residents? What would they most likely prefer?

Interview guideline relatives

**Narrative of a typical visit/GP visit in nursing homes**	We are interested in your experience. How is it during your visits in the nursing home normally? What are typical situations when you think of the care of your relative? Can you describe a typical home visit of the GP? How is the visit initiated? How does the medical care

	proceed usually?

**Description of the last GP visit**	Please remember the last home visit of a general practitioner in the nursing home. How was it in this particular case? Please describe the exact process. What happened afterwards?<br>**or if the relative was not present**: What did your relative tell you about the visit?
**Experience with areas of responsibility and distribution of task**	What tasks does your relative perform by himself? What are your tasks? How do GPs and nurses collaborate according to your experience?
**Ideas and phantasies about the ideal care in a nursing home**	Let your imagination run wild. What would be the ideal medical care in a nursing home for you?

Interview guideline residents

**Narrative of a typical day/ GP visit in nursing homes**	You have been living in the nursing home for quite a while. We are interested in your everyday experience. How does a normal day look for you? What are typical situations? Could you tell us about a typical GP´s visit? How is it initiated? How does the medical care proceed usually?
**Description of the last GP visit**	Please remember the last home visit of a general practitioner here in the nursing home. How was it in this particular case? Please describe the exact process.
**Experience with areas of**	We are interested in the distribution of tasks in a nursing home.

**responsibility and distribution of task**	What are the tasks and responsibilities of nurses and what are the tasks and responsibilities of the general practitioner? How do your relatives support you?
**Ideas and phantasies about the ideal care in a nursing home**	Let your imagination run wild. What would be the ideal medical care in a nursing home for you? What would you like to see there?
