# Supplementary material for: Interprofessional collaboration in nursing homes (interprof): development and piloting of measures to improve interprofessional collaboration and communication: a qualitative multicentre study
Source: BMC Fam Pract. 2018 Jan 11;19:14. doi: 10.1186/s12875-017-0678-1 (PMC5765653; doi:10.1186/s12875-017-0678-1)
Supplement: Supplementary file 2 — Interview guideline study part 3. (PDF 175 kb) [file 12875_2017_678_MOESM2_ESM.pdf]

Additional file 2

Interview guideline study part 3

Interview guideline pilot study interviews nurses and GPs

**Measures**	How did you experience the interprofessional collaboration since the implementation? Which experiences did you make? What has changed?<br>In which of the measures have you been involved actively?<br>How were the measures set in action? Was measure a,b,c… feasible?<br>Were there differences in the feasibility of the measures?<br>How useful was the measure a,b,c…in your daily work?<br>Where do you see the use of measure a,b,c…?<br>Which influence had measure a,b,c… on the provision of medical care of the residents – in your opinion?<br>If so, in what way did the residents realise the implementation of the measures?
**Interprofessional collaboration**	In your opinion- has there been a change in the quality of interprofessional collaboration due to the implementation of the measures? If yes, what has changed?<br>How did you experience the collaboration with the nurse/GP?
**Assessment**	What will happen to the measures from now on?<br>What could still be improved?<br>Which overall assessment would you give?<br>Is there something you would like to say additionally?
