# Supplementary material for: Interprofessional collaboration in nursing homes (interprof): development and piloting of measures to improve interprofessional collaboration and communication: a qualitative multicentre study
Source: BMC Fam Pract. 2018 Jan 11;19:14. doi: 10.1186/s12875-017-0678-1 (PMC5765653; doi:10.1186/s12875-017-0678-1)
Supplement: Supplementary file 3 — Interview quotations availability fax. (PDF 147 kb) [file 12875_2017_678_MOESM3_ESM.pdf]

Additional file 3

A selection of quotations with regard to availability/fax from the interviews

Nurses´ interviews

„ […] this morning I realised that the resident had a lot of mucus and couldn´t cough it up. Then I sent a fax to the GP. We work quite a lot with faxes. And I informed him about her general state and asked for a home visit which happened afterwards. CP04/4

“There are physicians […] who cannot be reached via telephone at all. To those I send a fax. […] wound so and so, grade so and so, it is like that. And currently we applied this. This is one kind of physicians. The others are actually reachable during specific telephone hours […]”. (AP2/72)

“[…] we fax quite a lot, we fax and we fax … and we fax.” (AP1/131-134)

“[…] a super GP, a real super GP, with whom you can really talk […] but the problem is, that we cannot reach him […]. Last Friday I sent him a fax […] because it was my opinion, that somebody needed an antibiotic. But until now I have no response from him, not Friday not Monday not now. When I call him: “We have not time, we have not time, send a fax”. Hmm this is real difficult […] I don´t know, what I should do now.” (AP2/74)

“Orders a placed via fax to the GPs. Everything just by fax because on the telephone information gets lost and via fax no GP can whitewash, he didn´t get it. Then we can always say: here is the fax with this date, with the signature and the confirmation of sending, and he cannot say, he didn´t receive it. This way we are on the safe side.“ (CP8/90)

GPs´ interviews

”Requests via telephone every day, I would say three to five. […] and ten to twenty requests via fax, which I answer in between.” (AA03 25)

“There are also nursing homes who fax their requests, and they know that I read them during lunch time, then they wait to see, if I call back, which I normally do. “ (BA03 /6)

"I send a fax: “no more injections”, I make the home visit, the resident still gets injections, that happens – or I say to my practice assistant: send a fax: “no more injections”, and the fax was not sent. Mistakes happen of course in communication, and they cannot be prevented, not without more effort bigger expenditure. Otherwise you must always confirm that you have received the information and understood it. This would be very very time consuming […]” (CA04/22-24)

”At the beginning, we were inundated with faxes […] then also the practice nurse didn´t know, that the other one sent a fax, partly double faxes, partly none […] we have organised it, that once a day they send a mail with all their requests[…]” (AA05/11)

”Yes, the good thing is, that because of the fax communication, the whole thing is easier to manage, when I send the prescription, then they can react on it, when they have time and I can react, when I have time.” (AA03/ 82)
