# Supplementary material for: Interprofessional collaboration in nursing homes (interprof): development and piloting of measures to improve interprofessional collaboration and communication: a qualitative multicentre study
Source: BMC Fam Pract. 2018 Jan 11;19:14. doi: 10.1186/s12875-017-0678-1 (PMC5765653; doi:10.1186/s12875-017-0678-1)
Supplement: Supplementary file 4 — Monoprofessional focus groups summary accessibility. (PDF 176 kb) [file 12875_2017_678_MOESM4_ESM.pdf]

Additional file 4

Accessibility of GP/nurse

Monoprofessional focus groups – summary of results Göttingen, Mannheim, Hamburg

Problem	Solution
**Accessibility of GP**<br>Massive efforts to reach GP via telephone<br>Only few opportunities to talk to GP<br>Processes in the GP´s practice delay the availability<br>Nurse has no influence on options in the practice (waiting for a call back, waiting to talk to the GP)	Immediate, reliable reactions to important issues<br>Set telephone hours<br>24 hours availability of GP/private mobile number<br>Communication via fax<br>Communication via internet (exchange of photos)<br>Communication via smartphone (face time, per video)<br>Better integration of the practice nurse
**Accessibility of nurse**<br>Only few opportunities to talk to the nurse	Communication via fax<br>One smartphone/ transportable telephone per floor<br>Cross shift contact person in nursing home
