# Supplementary material for: Interprofessional collaboration in nursing homes (interprof): development and piloting of measures to improve interprofessional collaboration and communication: a qualitative multicentre study
Source: BMC Fam Pract. 2018 Jan 11;19:14. doi: 10.1186/s12875-017-0678-1 (PMC5765653; doi:10.1186/s12875-017-0678-1)
Supplement: Supplementary file 5 — Interprofessional focus groups summary results fax. (PDF 189 kb) [file 12875_2017_678_MOESM5_ESM.pdf]

Additional file 5

Availability of GP (telephone, fax)

Interprofessional focus groups – summary of results from Göttingen, Mannheim, Hamburg

**Implementation:**<br>Agreement of communication path between practice and nursing home<br>Precondition: Both sides need to want it<br>Availability of GP in joint practice is higher	
**Telephone**<br>**….**	**Fax**<br>Mainly wish for communication via fax<br>Additional availability via telephone at fix times or in case of emergency<br>GPs "educate" nursing homes to use fax more
….	**Content**<br>Summary of telephone call (nursing home should fax results of telephone call to GP)<br>Results of the GP home visit<br>Forwarding of specific information<br>Request for a call back (GP)- (deescalation of situation often possible)<br>Using the label "urgent" leeds to a home visit
…	**Time**<br>Quick reaction via fax possible<br>Not time-consuming (quick to read, quick to answer)
…	**Advantages**<br>**GPs**<br>Fax for communication during practice opening hours<br>Documentation for legal purposes<br>Fax provides more options to decide<br>Nurses have more time to consider; no waste of time for GP<br>**Nurses**<br>Problem can be described immediately<br>Instructions from GP in written form<br>Fax is feasible<br>Fax fits better in working procedures<br>Signing of initials of nurses prevents redundant work<br>**Practice nurse**<br>Processes the fax requests from the nursing homes<br>Prioritisation of the requests, important requests on GP´s desk, less important into a filing basket for further processing<br>**Generally**<br>Fax as legal certainty, fax is easier for everybody
	**Disadvantages GP**<br>Irrelevant requests (only important to Medical Service of Health Insurers)<br>Waste of paper<br>Practice organization: GP does not want to be disturbed during opening hours
	**Wish: establishment of fax culture/obligatory processes**<br>Preparation of the home visit: nurses should fax to GP for a better preparation (number of residents, formalities, necessary devices)<br>Standardized fax forms from GP practice<br>Suggested prescriptions on the fax to ease the process in advance<br>If no fax arrives, the GP must not visit the nursing home<br>Faxes are more predictable, if a regular home visit day exists
	**Resources**<br>Availability of faxes to nursing station, fax with high resolution
