# Supplementary figures and images for: Interprofessional collaboration in nursing homes (interprof): development and piloting of measures to improve interprofessional collaboration and communication: a qualitative multicentre study
Source: BMC Fam Pract. 2018 Jan 11;19:14. doi: 10.1186/s12875-017-0678-1 (PMC5765653; doi:10.1186/s12875-017-0678-1)

Additional file 7  
expert workshop:  
availability- FAX

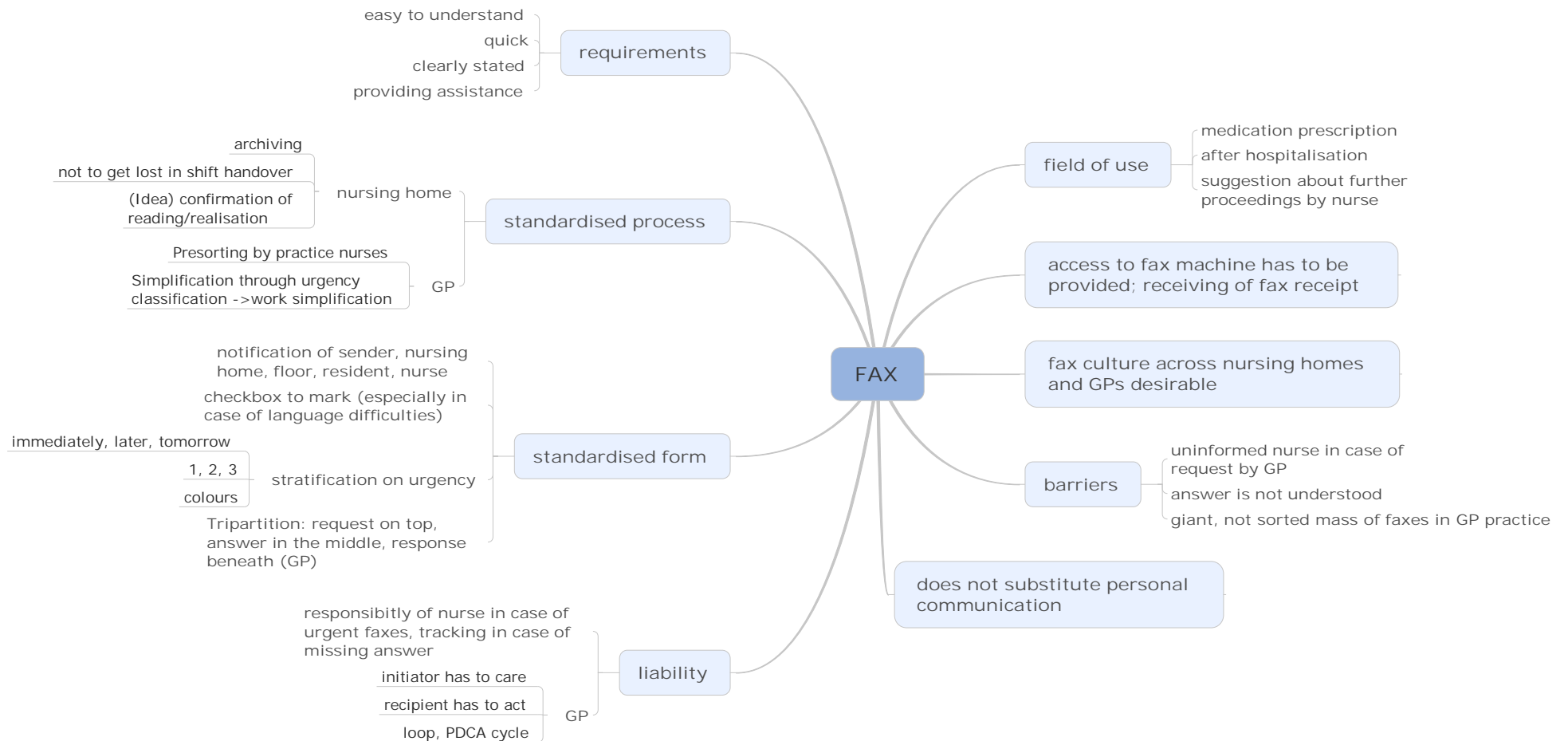

Supplement: Supplementary file 6 — Expert workshop results fax. (PDF 11 kb) [file 12875_2017_678_MOESM6_ESM.pdf]
