# Supplementary material for: Interprofessional collaboration in nursing homes (interprof): development and piloting of measures to improve interprofessional collaboration and communication: a qualitative multicentre study
Source: BMC Fam Pract. 2018 Jan 11;19:14. doi: 10.1186/s12875-017-0678-1 (PMC5765653; doi:10.1186/s12875-017-0678-1)
Supplement: Supplementary file 7 — Fax form. (PDF 197 kb) [file 12875_2017_678_MOESM7_ESM.pdf]

Additional file 7
Fax form

interprof

FAX

☐ urgent/ immediately ☐ during the next half day ☐ today ☐this week

resident: ____________________________ **date of birth:** ____________ **date, time FAX:**

nursing home: ____________________________ **GP:** ____________________________

floor:
nurse:
telephone number: until:

REQUEST/ NOTICE

☐medication ☐before home visit ☐ other

☐ please send confirmation of acknowledgement (when indicated without detailed answer)

☐ return confirmation acknowledgement **date, time FAX:**

ANSWER

☐ please send confirmation of acknowledgement (when indicated without detailed answer)

☐ return confirmation acknowledgement

ACTION

answer received, date, time: **initials:**

Comments

GEFÖRDERT VOM
Bundesministerium für Bildung und Forschung
